# Wearing a Bicycle Helmet Can Increase Risk Taking and Sensation Seeking in Adults

**DOI:** 10.1177/0956797615620784

**Published:** 2016-01-06

**Authors:** Tim Gamble, Ian Walker

**Affiliations:** Department of Psychology, University of Bath

**Keywords:** risk taking, sensation seeking, social priming, bicycling, protective equipment, behavior change, open data

## Abstract

Humans adapt their risk-taking behavior on the basis of perceptions of safety; this risk-compensation phenomenon is typified by people taking increased risks when using protective equipment. Existing studies have looked at people who know they are using safety equipment and have specifically focused on changes in behaviors for which that equipment might reduce risk. Here, we demonstrated that risk taking increases in people who are not explicitly aware they are wearing protective equipment; furthermore, this happens for behaviors that could not be made safer by that equipment. In a controlled study in which a helmet, compared with a baseball cap, was used as the head mount for an eye tracker, participants scored significantly higher on laboratory measures of both risk taking and sensation seeking. This happened despite there being no risk for the helmet to ameliorate and despite it being introduced purely as an eye tracker. The results suggest that unconscious activation of safety-related concepts primes globally increased risk propensity.

People’s perceptions of safety influence their risk taking. This phenomenon, studied under such rubrics as risk compensation (Adams & Hillman, 2001), risk homeostasis (Wilde, 1998), and risk allostasis (Lewis-Evans & Rothengatter, 2009), is typified by people taking increased risks when using protective equipment (Adams, 1982) or at least reducing their risk taking when protective equipment is absent (Fyhri & Phillips, 2013; Phillips, Fyhri, & Sagberg, 2011). Behavioral adaptation in response to safety equipment has been reported in studies examining drivers operating a vehicle with and without built-in safety devices (Sagberg, Fosser, & Sætermo, 1997), children running obstacle courses with and without safety gear (Morrongiello, Walpole, & Lassenby, 2007), and bicyclists descending a steep hill with and without helmets (Phillips et al., 2011). Work to date has been based on the assumption that people respond only to safety measures of which they are aware—an idea encapsulated in Hedlund’s first rule of risk compensation: “If I don’t know it’s there, I won’t compensate for a safety measure” (Hedlund, 2000, p. 87). Moreover, in research to date, the risk-taking behavior has been in the same domain as the safety measure (e.g., studies of seat-belt use in driving speed; Janssen, 1994).

Here, we changed both these approaches. First, we induced people to wear a helmet without their necessarily being aware they were wearing safety equipment: Participants were (falsely) told they were taking part in an eye-tracking study so we could exploit the fact that the head-mounted eye-tracking device we employed comes with both a bicycle helmet and a baseball cap as its standard mounting solutions. At random, participants were assigned to wear one mount or the other and were simply told it was the anchor for the eye tracker. Second, we divorced risk-taking behavior from the safety device by using a computerized laboratory measure called the Balloon Analogue Risk Task (BART; Lejuez et al., 2002), in which the helmet could do nothing to change risk. We also measured sensation seeking and anxiety as possible explanatory variables for any effect.

## Method

### Participants

Eighty participants (15 male and 24 female in the helmet condition, 19 male and 22 female in the cap condition) between the ages of 17 and 56 years (*M* = 25.26, *SD* = 6.59) took part in the study; no monetary reward was offered for participation. An a priori power analysis showed that 40 participants per condition should have 80% power to detect an effect size (Cohen’s *d*) of 0.63. This was deemed sufficient, as we hoped to see relatively substantial effects of the helmet manipulation.

### Materials

State anxiety was measured using the State-Trait Anxiety Inventory (STAI) Form Y-1 (Spielberger, 1983). This form contains 40 questions, 20 that measure a person’s feelings of anxiety right at the moment of response and 20 that measure his or her chronic levels of anxiety. Participants here answered the former set. In the BART (Lejuez et al., 2002), which we programmed in Real Studio (Xojo, 2011), participants pressed a button to inflate an animated balloon on a computer screen. Each button press inflated the balloon more and increased the amount of fictional currency earned. If the balloon burst (which it would at a random point between 1 and 128 inflations), all earnings for that trial were lost. At any point, participants could choose to stop pumping and “bank” their accrued money. After the balloon burst, or after a decision to bank, the next trial began. Each participant completed 30 trials, and his or her risk-taking score was the mean number of pumps made on trials on which the balloon did not burst. This score would be higher when participants risked losses by trying to maximize their score and lower when participants avoided risk and played more conservatively.

Sensation seeking was measured using the Sensation-Seeking Scale Form V (Zuckerman, Eysenck, & Eysenck, 1978). This scale measures four dimensions (10 self-report items each) of sensation-seeking behavior: thrill and adventure seeking, disinhibition, experience seeking, and boredom susceptibility. Bicycling frequency was measured using a Likert scale ranging from 1 (*never*) to 6 (*five times a week or more*). If a person selected anything other than “never” on this instrument, helmet-wearing frequency was measured using a Likert scale ranging from 1 (*never*) to 6 (*always*).

**Fig. 1.**
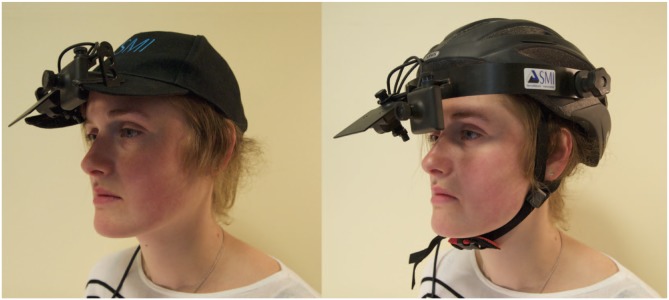
Photos showing how the eye tracker was mounted in each of the two conditions: to a baseball cap (left) and a bicycle helmet (right).

Either an Abus (Phoenix, AZ) HS-10 S-Force peak bicycle helmet or a Beechfield (Bury, United Kingdom) B15 five-panel baseball cap was used to support the SensoMotoric (Teltow, Germany) head-mounted iView X HED-4.5 eye-tracking device (with its delicate 45° mirror removed; see Fig. 1). Participants responded to the scales using the Bristol Online Surveys Web site. All measures and the BART were completed on a 19-in. 4:3 LCD monitor. The experimenter “operated” an Applied Science Laboratories (Bedford, MA) Eye-Trac 6 desk-mounted optics system with Eye-Trac PC. A fake nine-point eye-tracking calibration program was written in Real Studio to increase the verisimilitude of the eye-tracking procedure.

### Procedure

This study was conducted in the University of Bath Department of Psychology’s eye-tracking laboratory. Participants were brought into the laboratory and told that they would complete a number of computer-based risk-taking measures while their point of gaze was measured using a head-mounted eye tracker. After reading information about the study on the computer screen and agreeing to participate, they entered their age and gender and completed the STAI Y-1. A screen then appeared saying that the eye tracker would now be set up; the experimenter placed the cap- or helmet-mounted eye tracker on the participant’s head, making a show of carefully aligning everything as in a real eye-tracking procedure. The experimenter then moved to the eye-tracking computer, where he or she ran the fake calibration software and conspicuously adjusted the eye-tracking controls to make it appear to participants that their eye movements were really being tracked. Participants then completed the Sensation-Seeking Scale, the BART, and the STAI Y-1 again. Afterward, a screen appeared saying that the eye tracker was to be turned off, and the experimenter removed the apparatus from participants’ heads. Participants then completed the final STAI Y-1 before being debriefed, at which point they were informed of the deception and asked not to share details of the experiment with anyone else. They then reported their bicycling frequency and, if they did cycle, their helmet-wearing frequency.

## Results

**Fig. 2.**
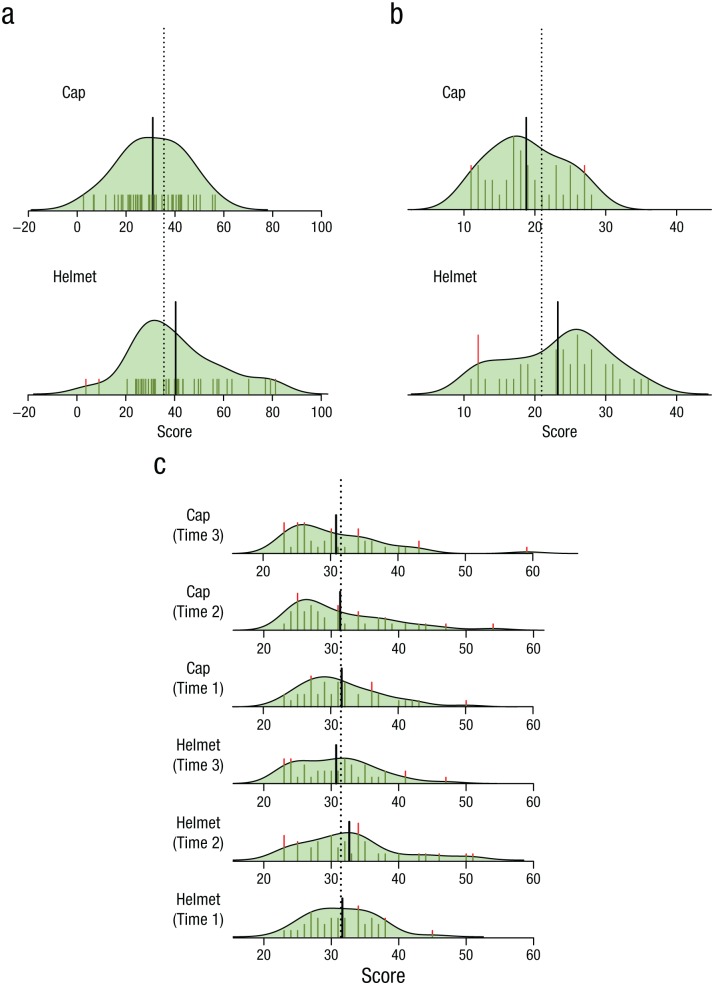
Distribution of scores for the helmet and cap conditions on (a) the Balloon Analogue Risk Task (BART), (b) the Sensation-Seeking Scale, and (c) state anxiety, measured using the State-Trait Anxiety Inventory (STAI). For anxiety, scores are shown separately for time points before donning the eye tracker (Time 1), while wearing the eye tracker (Time 2), and after removing the eye tracker (Time 3). For each measure, the mean score across conditions is indicated by a vertical dotted line, and the mean score for each condition separately is indicated by a thick vertical line. Individual participants’ scores are shown as thin vertical lines (rug points; stacked when more than 1 participant obtained the same score). Overlaid on the rug-point plots are kernel-density curves (with arbitrary scaling) that illustrate the overall distribution of scores within each condition.

Wearing a helmet was associated with higher risk-taking scores (*M* = 40.40, *SD* = 18.18) than wearing a cap (*M* = 31.06, *SD* = 13.29), *t*(78) = 2.63, *p* = .01, *d* = 0.59 (Fig. 2a). Similarly, participants who wore a helmet reported higher sensation-seeking scores (*M* = 23.23, *SD* = 7.00) than participants who wore a cap (*M* = 18.78, *SD* = 5.09), Welch’s *t*(69.19) = 3.24, *p* = .002, *d* = 0.73 (Fig. 2b). These effects cannot be explained by the helmet affecting anxiety, as anxiety did not change significantly as a function of condition, *F*(1, 78) = 0.19, *p* = .66, time of measurement, *F*(2, 156) = 2.37, *p* = .10, or an interaction between the two, *F*(2, 156) = 1.18, *p* = .31 (Fig. 2c). Note that we used the square roots of the anxiety scores for analyses because of the skew seen in Figure 2c. There was no relationship between risk taking and gender, *t*(78) = 0.45, *p* = .66, bicycling experience (ρ = .12, *p* = .27), and extent of helmet use when bicycling (ρ = .06, *p* = .60), nor, in regression modeling, interactions of any of these variables (e.g., the Condition × Bicycling Experience interaction was not significant; *t* = 0.39, *p* = .70). Prior research has shown that helmets do not affect cognitive performance in demanding laboratory tasks (Bogerd, Walker, Brühwiler, & Rossi, 2014), which means the results cannot be attributed to this factor either.

## Discussion

Laboratory measures showed greater risk taking and sensation seeking when participants wore a helmet, rather than a baseball cap, during testing. These effects arose even though the helmet was introduced as a mount for an eye-tracking apparatus and not as safety equipment, and even though it could do nothing to alter participants’ level of risk on the experimental task. Notably, the effect was an immediate shift in both risk taking and sensation seeking. This finding contrasts with those of previous work on unconscious influence, such as experiments on the persuasive effects of head movements (Wells & Petty, 1980) and environmental cues on consumer behavior (Berger & Fitzsimons, 2008), which looked instead at longer-term attitudinal changes from more overt signals.

Our findings are plausibly related to social priming, wherein social behaviors are cued by exposure to stereotypes or concepts (Bargh, 2006). However, whereas social priming is generally understood in terms of behavior directed toward another person, the effects in this study were individual, focused on the risk-taking propensity of a person acting alone during exposure to a safety-related prime. Schröder and Thagard (2013) produced computational models of social priming in which primes activate shared cultural concepts in people’s minds, which in turn are associated with actions; through these links, the actions become available to the behavioral selection process. Speculatively, if what we saw in this study were to be understood through such mechanisms, with the helmet invoking concepts of protection from risk and thereby subconsciously shaping behaviors, our findings might suggest that Schröder and Thagard’s social-priming framework operates even when its interaction target component (another person with whom to interact) is absent.

Our findings initially appear different from those of some other studies. Fyhri and Phillips (2013; Phillips et al., 2011) found that risk taking in downhill bicycling, measured through riding speed, did not simply increase when a helmet was worn; rather, the people who normally cycled with a helmet took fewer risks when riding without one. Why did the participants in Fyhri and Phillips’s study who were not habitual helmet users not react to wearing a helmet with increased risk taking, as our experiment might suggest they would? Clearly more work is needed to definitively pin down all the mechanisms here, but for now, we speculate that the difference might be related to considerable variations between the two studies’ procedures. Fyhri and Phillips greatly emphasized the physicality of their task (“to increase the difference in measures between the helmet-on and -off conditions, all participants were instructed to cycle using one-hand in both conditions”; p. 60), which provides a direct link between the action (bicycling) and the condition (helmet wearing) that was absent in our study. Moreover, that study used a repeated measures design, in which participants were aware they were riding a bicycle both with and without a helmet. This could have meant that behavior changed through mechanisms different from those seen here, where participants took part only in one condition and were not aware of any manipulation, nor even that they were specifically wearing a safety device.

The practical implication of our findings, in which risk taking changed in a global way when the helmet was worn, might be to suggest more extreme unintended consequences of safety equipment in hazardous situations than has previously been thought. The idea that people might take more risks when wearing safety equipment designed to protect against those risks has a considerable (Adams, 1982, 1995; Adams & Hillman, 2001; Hedlund, 2000), although not uncontroversial (McKenna, 1988), history. If this laboratory demonstration of globally increased risk taking arising from localized protection were to be replicated in real settings, this could suggest that people using protective equipment against specific hazards might also be unduly inclined to take risks that such protective equipment cannot reasonably be expected to guard against. This is not to suggest that the safety equipment will necessarily have its specific utility nullified, but rather that there could be changes in behavior wider than previously envisaged.

## Supplementary Material

Supplementary material
